# Modern Methods of Obtaining Synthetic Oil from Unconventional Hydrocarbon Raw Materials: Technologies, Catalysts, and Development Prospects

**DOI:** 10.3390/polym17060776

**Published:** 2025-03-14

**Authors:** Aisha Nurlybayeva, Ainura Yermekova, Raushan Taubayeva, Nurbanu Sarova, Ardak Sapiyeva, Sulushash Mateeva, Gulsim Matniyazova, Kamila Bulekbayeva, Gulim Jetpisbayeva, Marzhan Tamabekova

**Affiliations:** 1 Department of Chemistry and Chemical Technology, M.Kh. Dulaty Taraz University, Taraz 080000, Kazakhstan; rustem_ergali@mail.ru (A.N.); mateeva73@mail.ru (S.M.); nurhat2000@mail.ru (K.B.); gulim_86@mail.ru (G.J.); 2 Department of Chemistry, M.Kh. Dulaty Taraz University, Taraz 080000, Kazakhstan; raushan.taubaeva@mail.ru; 3 Department of Chemistry, Kazakh National Medical University named after S.D. Asfendiyarov, Almaty 050012, Kazakhstan; sarova.n@kaznmu.kz; 4 Department of General and Biological Chemistry, Astana Medical University, Astana 010000, Kazakhstan; 5 Higher School of Natural Sciences, Astana International University, Astana 010000, Kazakhstan; gulsim.matniyazova@mail.ru; 6 School-Gymnasium 97, Astana 020000, Kazakhstan; marzhan1991@mail.ru

**Keywords:** synthetic oil, unconventional hydrocarbon feedstock, pyrolysis, catalytic depolymerization, waste economics, environmental aspects, energy security

## Abstract

This article considers modern approaches to obtaining synthetic oil from unconventional hydrocarbon feedstocks, including plastic waste, tires, biomass, coal, and extra-heavy oil. Particular attention is paid to multi-stage technologies, such as pyrolysis, catalytic depolymerization, gasification followed by Fischer–Tropsch synthesis, and hydrocracking of heavy residues. The important role of catalysts in increasing the selectivity and economic efficiency of processes is noted: nanostructured, bifunctional, and pollution-resistant systems are increasingly used. Economic factors influencing the competitiveness of this industry are considered, including the volatility of prices for traditional oil, government support measures, and the development of waste logistics infrastructure. It is emphasized that the strengthening of the position of synthetic oil is associated with the growth of environmental requirements stimulating the recycling of plastics, tires, and biomass; at the same time, compliance with high environmental standards and transparency of emission control play a critical role in the social aspects of projects. In addition to improving the environmental situation, the development of synthetic oil contributes to the creation of jobs, the resolution of problems of shortage of classical oil fields, and the increase of energy security. It is concluded that further improvement of technologies and integration into industrial clusters can turn this sphere into a significant component of the future energy sector.

## 1. Introduction

The modern energy industry faces serious challenges associated with the depletion of natural reserves of traditional oil. This resource has been the main source of energy and raw materials for many industries for decades, providing the production of fuel, plastics, chemicals, and pharmaceuticals. However, according to experts, easily extractable oil deposits are gradually being depleted, and hydrocarbon consumption continues to grow. In these conditions, the world community is faced with the task of finding alternative sources of hydrocarbon raw materials and developing new technologies for their processing.

One of the most promising solutions is the production of synthetic oil from unconventional carbon-containing materials. Synthetic oil is a liquid hydrocarbon that can be obtained from sources such as shale oil, bitumen sands, coal, biomass, and carbon-containing waste. These resources are more widely available than traditional oil and can become the basis for the production of fuels and chemicals in the future.

The relevance of developing technologies for producing synthetic oil is due to several important factors. Firstly, the transition to the use of unconventional raw materials allows for diversifying hydrocarbon sources and reducing dependence on natural oil deposits. Secondly, it helps ensure energy independence for countries that do not have significant oil reserves but have alternative resources such as coal or biomass. Thirdly, the use of carbon-containing waste to produce synthetic oil helps solve environmental problems such as the accumulation of plastic waste and carbon dioxide emissions.

The transition to the use of unconventional hydrocarbon raw materials is becoming not just an economic necessity but also an environmental challenge. The use of waste and biomass processing technologies helps reduce environmental pollution and reduce the carbon footprint, which is especially important in the context of combating global climate change.

Modern methods of producing synthetic oil include several basic technological processes, such as pyrolysis, hydrocracking, gasification, and Fischer–Tropsch synthesis. These methods allow for the conversion of carbon-containing raw materials into liquid hydrocarbons with various characteristics. Catalysts play an important role in these processes, significantly increasing the efficiency of raw material conversion and improving selectivity for obtaining target products.

The development of synthetic oil production technologies is also associated with global economic and political trends. For many countries, it is becoming important to reduce dependence on oil imports and create their own fuel production based on local resources. This is especially true for countries that have significant reserves of coal, shale gas, biomass, and carbon-containing waste. In addition, synthetic oil can be used as a more environmentally friendly fuel, which is important in the context of the transition of the world economy to reducing carbon emissions.

Thus, the production of synthetic oil from unconventional hydrocarbon raw materials is a promising direction for the development of modern energy and chemical industries. It allows for solving economic, environmental, and political problems simultaneously.

This article is devoted to the consideration of modern methods of obtaining synthetic oil, the analysis of technologies and catalysts that are used in these processes, and the prospects for their implementation in industrial production.

## 2. Classification of Unconventional Hydrocarbon Resources for Oil Synthesis

**Figure 1:**
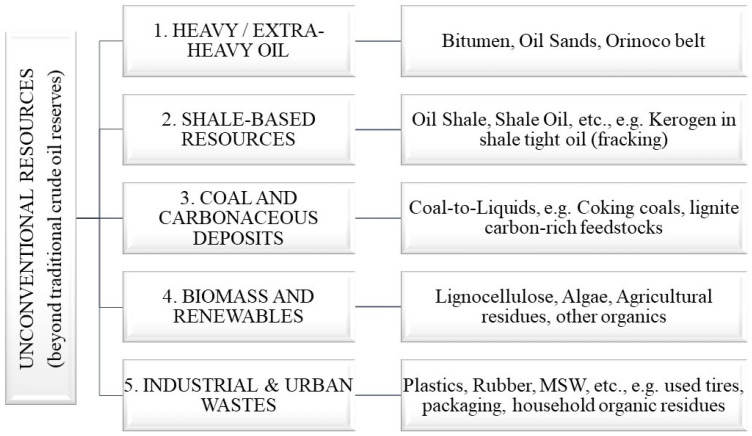
Unconventional hydrocarbon resources for oil synthesis.

Unconventional hydrocarbon resources are alternative sources of raw materials that can be used to produce synthetic oil. Unlike traditional oil extracted from underground deposits, these resources have a more complex structure and require the use of special processing technologies. The classification of unconventional hydrocarbon raw materials includes several main groups that differ in origin, composition, and processing methods [1,2,3,4]. Classification of unconventional hydrocarbon resources for oil synthesis is shown in Figure 1.

### 2.1. Oil Shale and Bituminous Sands

Oil shale is one of the most promising sources of hydrocarbon raw materials used to produce synthetic oil. These are sedimentary rocks containing organic matter, kerogen, which decomposes into hydrocarbons when heated and can be processed into liquid fuel. Unlike traditional oil and gas, oil shale requires more complex hydrocarbon extraction technology and high energy costs for processing. However, the rich reserves of oil shale around the world make it an important resource for the energy industry, especially in the context of depleting traditional oil fields [5].

Oil shale has a high mineral content (up to 90%) and a relatively low organic matter content (from 10 to 40%). The main part of the rock is a mineral matrix, including clay minerals, quartz, carbonates, and sulfates. The organic part is represented by kerogen—a high molecular compound containing carbon, hydrogen, oxygen, nitrogen, and sulfur [6]. Kerogen is a precursor of hydrocarbons and, under the influence of high temperatures, is converted into liquid and gaseous hydrocarbons.

According to the chemical composition, kerogen in oil shale is divided into three types:

- Type I contains mainly lipids and has a high potential for oil production.
- Type II is a mixed type consisting of hydrocarbons and carbohydrate residues, suitable for obtaining both oil and gas.
- Type III is characterized by a high carbohydrate content and a low lipid content, mainly used to obtain gas.
- Large deposits of oil shale are located in the USA, Estonia, China, and Brazil. For example, Estonia actively uses oil shale raw materials to produce electricity and synthetic fuel [7].
- Despite the significant potential of oil shale, its processing is an energy-intensive process and has a negative impact on the environment [8]. The main environmental problems are associated with the formation of large amounts of waste and carbon dioxide emissions.
- The economic efficiency of oil shale processing depends on oil prices and the technical level of the developed technologies. In the context of high oil prices, interest in shale projects is increasing, which stimulates the development of new processing methods.

*Bituminous sands* are one of the most significant types of unconventional hydrocarbon raw materials that can serve as a source of synthetic oil. Their main feature is that these sand deposits contain bitumen, an extremely viscous and heavy form of oil [9]. In order to turn such bitumen into usable liquid fuel, special extraction and processing technologies are needed that take into account both the physical and chemical properties of this material.

Bituminous sands can be characterized as a mixture of quartz sand, water, clay, and bitumen. The latter is the key component since it is from bitumen that synthetic oil is obtained during processing. At the same time, the concentration of bitumen usually does not exceed 10–15% of the total volume of the rock, and the rest is sand, clay, and water. The high density and viscosity of bitumen hinder its free outflow, as a result of which it cannot move in the thickness of the earth’s crust, like traditional oil [10].

The largest deposits of bituminous sands are concentrated in Canada, Venezuela, and Russia. It is in Canada that bitumen sand development is at its most advanced stage of technological development where it occupies an important place in the structure of oil production and the production of synthetic oil [11].

Depending on the depth and density of the deposits, two main methods of extracting bituminous sands are used: surface mining (open-pit mining) and underground extraction (in situ methods) [12]. A surface mining method is used where the bitumen sand layers are not very deep—usually up to 75 m below the surface. The rock is opened using excavators, then the sand is transported to processing plants. Here, hot water extraction is used: when in contact with hot water, the bitumen liquefies, separates from the sand, and floats to the surface, forming an emulsion of water and hydrocarbons [13].

If the bituminous sand layers are deeper, the technology of heating the rock with steam or solvents directly in the layers is used. One of the most common methods is SAGD (steam-assisted gravity drainage), which involves injecting hot steam into the formation, reducing the viscosity of the bitumen, and allowing it to flow to the bottom of the formation, from where it is pumped to the surface. Alternative methods include the use of solvents (for example, a mixture of steam and light hydrocarbons) or electric heating technology. Underground methods help avoid large-scale disturbance of the landscape and reduce the volume of tailings storage facilities, unlike open-pit mining, but they require large energy costs and complex engineering solutions [14,15].

The processing of bituminous sands, despite the high potential for obtaining synthetic oil, is associated with a number of environmental problems. Among the main negative factors are high water consumption, especially in surface mining; high greenhouse gas emissions due to the intensive use of energy to heat the formations or water; and significant transformation and degradation of the landscape in open mining, including the formation of tailings containing toxic waste.

### 2.2. Biomass

Biomass is a wide range of organic materials of plant and animal origin that can serve as an alternative source of hydrocarbon raw materials. In the context of depleting traditional oil reserves and a growing environmental crisis, the processing of biomass into synthetic oil is becoming increasingly important. High reproducibility, availability, and diversity of biological resources make biomass one of the most promising areas for the development of modern technologies for obtaining fuel and chemical raw materials [16].

Biomass is usually understood as any organic matter formed by living organisms. This includes agricultural waste (straw, corn cobs, seed husks), wood residues and sawdust, algae, household organic waste, and some types of industrial organic waste. The diversity of biomass composition determines the different methods of its conversion into liquid hydrocarbons [17].

In chemical terms, biomass is rich in cellulose, lignin, and hemicellulose, as well as various extractive substances (fats, resins). This determines its high energy potential: during thermal processing, these compounds can be converted into gaseous and liquid fractions, similar in composition and properties to traditional oil [18].

Using biomass to produce synthetic oil has a number of significant advantages. First, biomass is a renewable resource as plant material and organic waste can be generated in large quantities with proper farming and rational use of land. Second, processing biomass promotes more rational waste management, reducing the load on landfills and dumps. Third, the carbon contained in biomass was recently absorbed from the atmosphere through photosynthesis, which provides the potential to neutralize or reduce the carbon footprint compared to fossil fuels [19].

However, there are also significant limitations. These include the need for preliminary preparation and sorting of raw materials, a relatively low yield of liquid hydrocarbons compared to some types of fossil fuels, and the complexity of the technological equipment. In addition, growing biomass on an industrial scale can compete with agricultural food production, and irrational use of land can lead to a loss of biodiversity.

### 2.3. Coal

Coal has long been considered a vital energy resource, providing humanity with heat and electricity since the Industrial Revolution. However, in the modern world, where traditional oil fields are depleted and the need for liquid fuels and chemical raw materials is still high, coal is considered not only as a fuel for power plants but also as a promising non-traditional hydrocarbon raw material from which synthetic oil can be obtained [20].

Coal is formed as a result of a long and complex process of accumulation and transformation of plant biomass under the influence of elevated temperature and pressure in the earth’s crust. Depending on the degree of coalification—that is, the depth of geological transformation of organic matter—there are several types of coal: brown, hard, and anthracite. The higher the degree of coalification, the higher the carbon content and energy value of the coal [21].

From a chemical point of view, coal is a solid mixture of high molecular organic substances, mainly of an aromatic structure, with inclusions of mineral impurities. Its organic part is represented by a complex conglomerate of macromolecules containing carbon, hydrogen, and oxygen, as well as small amounts of nitrogen and sulfur [22].

The key advantage of coal as an unconventional hydrocarbon raw material is its vast global reserves. Many countries that do not have significant oil reserves have large coal deposits, which potentially provides them with raw material independence. In addition, gasification and direct hydrogenation technologies, which have been perfected for decades, can produce high-quality fuel, almost as good as traditional oil.

However, the use of coal for oil synthesis is also associated with a number of problems. First of all, the processes of converting coal into liquid hydrocarbons require significant energy costs, which inevitably affects the overall economic feasibility. High energy intensity and the need for expensive equipment and catalysts make coal liquefaction projects competitive only at relatively high oil prices.

Another serious problem is the impact on the environment [23]. Coal mining, especially open-pit mining, negatively affects the landscape and ecosystems, and processing is accompanied by emissions of carbon dioxide and other pollutants. The development of environmentally friendly carbon capture and storage (CCS) methods could alleviate this problem, but its use adds to the already high cost of the production cycle.

### 2.4. Industrial and Household Waste

In the modern world, the topic of waste recycling is increasingly intertwined with issues of energy security and resource conservation. Industrial and household waste, accumulated over many years in huge volumes, is today increasingly considered as a potential raw material for obtaining a valuable product—synthetic oil. Such recycling not only partially solves the problem of waste disposal, but also contributes to the formation of alternative hydrocarbon resources that can supplement or replace traditional oil [24].

The totality of industrial and household waste includes a wide range of materials: plastic products (bottles, packaging, toys), rubber and tires, textiles, food scraps, paper, and wood trimmings, as well as various organic and inorganic substances. In the context of oil synthesis, the most valuable in terms of energy potential are wastes containing a significant proportion of carbon and hydrogen, such as plastics and rubber. Their chemical composition makes it possible to process them into liquid hydrocarbons at appropriate temperatures and conditions.

Plastic waste (PET, HDPE, LDPE, polypropylene, etc.) and rubber are synthetic polymers with many carbon–hydrogen bonds in their structure. It is due to this that their thermal processing allows us to obtain initial hydrocarbon fractions, which can subsequently be converted into liquid fuel or components for the petrochemical industry [25].

Processing industrial and household waste into synthetic oil can make a significant contribution to solving environmental problems and the rational use of resources. Firstly, waste disposal in this way reduces the load on landfills and dumps, minimizing the pollution of soil and water resources. Secondly, processing materials with a high carbon and hydrogen content makes it possible to reduce the consumption of fossil raw materials, thereby supporting the concept of a “closed cycle” and the transition to a more environmentally friendly economy [26].

However, questions remain about the profitability of these technologies. Despite the obvious advantages, pyrolysis and gasification processes require high energy costs, as well as expensive equipment and catalysts [27]. The economic efficiency of such installations largely depends on oil prices, government subsidies, and environmental legislation. Under favorable conditions, such projects can become profitable investments, contributing to the development of a “green” economy and reducing carbon dioxide emissions.

In the context of growing global hydrocarbon consumption and increasing volumes of accumulated waste, waste-to-liquid fuel processing technologies have significant potential. The development of catalysts and process schemes, automation, and scaling of production allow us to hope for a further reduction in the cost of the final product. In addition, increasing environmental requirements aimed at reducing greenhouse gas emissions and solving the problem of plastic in the oceans are stimulating researchers and entrepreneurs to search for new solutions.

### 2.5. Potential of Feedstock for Producing Synthetic Oil

The variety of unconventional hydrocarbon sources opens up a wide range of possibilities for producing synthetic oil, although each type of feedstock has its own characteristics, advantages, and limitations [28]. Below is an overview of the potential of the main groups of unconventional hydrocarbon feedstocks.

Table 1 shows the potential of each type of feedstock for producing synthetic oil.

Thus, each type of non-traditional raw material has its own set of advantages (reserves, availability, environmental benefits) and limitations (technological difficulties, high costs, environmental risks). The optimal choice in favor of one or another raw material is determined by the balance of economic feasibility, technological maturity, and environmental consequences.

## 3. Technologies for Producing Synthetic Oil

In the modern world, where traditional oil fields are gradually being depleted, the use of non-traditional hydrocarbon resources is becoming increasingly relevant. A number of technological processes have been developed for processing such raw materials, be it oil shale, bitumen sands, biomass, coal, or waste.

### 3.1. Pyrolysis

Pyrolysis is a promising technology for converting unconventional raw materials into synthetic oil, leveraging thermal decomposition in the absence of oxygen [29]. Pyrolysis is rightfully considered one of the most universal methods of thermal processing of organic materials, capable of converting almost any carbon-containing raw material into liquid and gaseous hydrocarbons with properties similar to oil and associated petroleum gas. The essence of this process is heating the feedstock to high temperatures (usually from 400 to 600 °C and higher) without oxygen [30]. As a result, large molecules of organic compounds—be they polymers, cellulose, or kerogen in oil shale—are destroyed and disintegrate into simpler substances. The main products in this case are the liquid fraction (pyrolysis oil or tar), gaseous components, and a solid residue, which is usually called coke or carbonaceous material [31].

The scope of pyrolysis is difficult to cover in one list because it is suitable for a wide range of raw materials. For example, it is used to process plastic waste—bottles, packaging, old tires, as well as biomass in the form of agricultural and forest residues. In the case of oil shale, shale oil is released during heating, which can then be further purified to a state close to traditional oil. Pyrolysis is also used for oil sludge, which is a heavy residue of oil production and processing. The pyrolysis mechanism is based on the rupture of chemical bonds at high temperatures. When oxygen does not enter the reactor, the organic material does not burn but rather decomposes into lighter substances. The liquid phase, which is obtained by cooling volatile products, usually consists of a large number of different compounds—from light hydrocarbons to complex oxygen-containing compounds. The gas mixture most often includes methane, hydrogen, carbon monoxide, and carbon dioxide. The solid residue remaining in the reactor is coal or coke [32,33].

There are two main ways of carrying out pyrolysis. Fast pyrolysis, which lasts only a fraction of a second, allows for the maximum yield of the liquid phase, called pyrolysis oil. This approach is especially in demand when the ultimate goal is to obtain liquid hydrocarbons, from which fuel or petrochemical products can then be synthesized. Slow pyrolysis takes longer, and the proportion of solid residue increases, which is most often used to produce charcoal and has less to do with the production of liquid fuel.

Pyrolysis oil itself is not always suitable for direct use in engines or petrochemical plants. To bring its properties closer to classic oil, it is necessary to carry out a number of additional operations. First of all, this can be hydrotreating—saturating pyrolysis oil with hydrogen and removing sulfur, nitrogen, and oxygen from it. Then the product is distilled, separating into fractions, each of which can undergo cracking or isomerization, if it is necessary to further improve the quality indicators.

Pyrolysis occurs in several stages: first, molecular bonds are broken to form radicals, then dehydrogenation and depolymerization reactions occur, leading to the formation of liquid and gaseous hydrocarbons, and in the final stage, undesirable coking processes are possible [34].

The use of catalytic pyrolysis allows for reducing the decomposition temperature and increasing the selectivity of liquid fractions formation. Zeolites (ZSM-5, Y-zeolites), metal oxide systems (NiO, ZnO, TiO_2_), and bifunctional catalysts combining acid and metal active centers are used for this purpose. The optimal catalyst should provide a high yield of liquid fuel, minimal coke formation, and stability of activity after repeated use [35].

Pyrolysis has significant advantages. It allows for the recycling of huge volumes of hard-to-decompose waste (plastic, rubber), turning it into a valuable hydrocarbon resource [36]. In addition, the technology is not tied to any one type of raw material and can be adapted to different production tasks and regions. However, the success of pyrolysis projects is largely determined by their economic profitability and impact on the environment. Maintaining the high temperatures required for the process requires large energy costs, and the diversity of the component composition of the raw materials necessitates complex purification of the resulting gases and liquids. When processing plastics and rubber, toxic substances are formed that must be effectively captured so as not to harm the environment and human health.

Despite the difficulties, the demand for pyrolysis as a technology will only grow as the modern world strives for more efficient waste management and the search for alternative sources of raw materials for the production of fuel and petrochemical products. Where the cost of traditional oil is high and there is a large volume of accumulated plastic and organic waste, pyrolysis can become a profitable and environmentally sound solution, forming a promising direction for the development of resource-saving energy.

### 3.2. Gasification with Subsequent Synthesis (Fischer–Tropsch Method)

Gasification with subsequent synthesis by the Fischer–Tropsch method is one of the most flexible and universal processes that allows for obtaining liquid hydrocarbons from a wide range of feedstocks. First of all, within the framework of this approach, the feedstock—coal, biomass, industrial or household waste, as well as oil refining residues—is subjected to high-temperature processing with limited oxygen access. The result is synthesis gas, consisting mainly of carbon monoxide (CO) and hydrogen (H_2_) [37].

Different types of reactors are used for gasification. In some cases, a boiling or fixed bed is used, in others—a special flow (enthalpy) gasifier where high temperatures (from 800 to 1500 °C) contribute to the decomposition of solid or heavy feedstock into a gas mixture. It is extremely important to ensure the correct conditions—temperature, pressure, and ratio of fuel and oxidizer (or water vapor)—in order to obtain gas with an optimal ratio of CO and H_2_. Often after primary gasification, the synthesis gas undergoes additional purification and composition adjustment: sulfur and other impurities are removed, and excess CO is converted into hydrogen using the water–gas shift reaction to achieve the desired balance for the subsequent catalytic reaction.

Then comes the Fischer–Tropsch synthesis itself, named after the German chemists who developed the process in the 1920s. In a specially designed reactor at elevated temperature and pressure (usually 200–350 °C and 10–40 bar) with the participation of a catalyst (usually iron-containing or cobalt-based), CO and H_2_ molecules are combined into more complex chains. The result is long-chain hydrocarbons, which, when cooled and fractionated, yield a wide range of liquid products—from light fractions to waxy substances resembling paraffin. After additional processing and purification, these substances are converted into diesel fuel, gasoline, aviation kerosene, or synthetic oil with a low sulfur content and excellent performance characteristics [38,39,40].

Gasification is a process of thermal conversion of carbon-containing raw materials (coal, biomass, plastics) into synthesis gas (CO + H_2_) at temperatures of 700–1200 °C. The resulting gas is used to synthesize liquid hydrocarbons using the Fischer–Tropsch method, during which carbon monoxide and hydrogen react on a catalyst to form long-chain hydrocarbons [41].

Catalysts based on iron (Fe), cobalt (Co), and ruthenium (Ru) are used for this process, with cobalt systems demonstrating high efficiency in low-temperature synthesis, and iron systems working better in conditions rich in CO_2_. Important efficiency criteria are the optimal CO:H_2_ ratio (~1:2), high carbon conversion, and catalyst stability over several reaction cycles [42].

The main advantage of this scheme is its versatility in the choice of raw materials [43]. Gasification allows for the processing of virtually any carbon-containing materials: coal, forest and agricultural waste, garbage, and even oil refining waste. This allows for the production of synthetic hydrocarbons in regions where there is a shortage of traditional oil, but significant reserves of alternative raw materials exist. The resulting fuel often surpasses traditional oil products in quality since it contains virtually no sulfur or nitrogen, and the cetane number of diesel fractions is quite high.

The process is not without its difficulties. Firstly, gasification units and Fischer–Tropsch reactors require significant capital investment [44]. The technology is complex, and the equipment must withstand high temperatures, pressure, and aggressive environments. Secondly, without government support or high oil prices, such projects can be economically risky since energy costs and expenses for gas purification and carbon dioxide capture (if provided) are significant. Finally, when processing large volumes of raw materials, the issue of by-product disposal arises, especially in waste gasification where the composition and properties of the feedstock can vary greatly.

Nevertheless, in the context of the gradual depletion of traditional oil fields and growing attention to environmental safety issues, the technology of gasification with subsequent Fischer–Tropsch synthesis demonstrates its potential and demand. It makes it possible to obtain high-quality fuel from almost any carbon feedstock, and, therefore, can serve as one of the pillars of energy independence and diversity. In addition, further improvement of catalysts and the development of more advanced CO_2_ capture and utilization schemes, as well as integration with renewable energy, can make this method even more efficient and environmentally justified.

### 3.3. Hydrocracking

Hydrocracking is rightfully considered one of the most popular technologies in the field of deep processing of hydrocarbon raw materials. It makes it possible to obtain high-quality, easily evaporated fractions from heavy and viscous components that are usually not suitable for direct processing. Initially, hydrocracking was in demand in the oil refining industry for the processing of tar, fuel oil, and other heavy residues of traditional oil. However, with the development of technology and the emergence of interest in unconventional resources—for example, shale oil, bitumen sands, and pyrolysis oil from waste—hydrocracking began to be considered a universal method for obtaining synthetic oil from a wide variety of hydrocarbon streams.

The peculiarity of hydrocracking is that the cracking process—the splitting of long molecules into shorter ones—occurs simultaneously with the saturation of the resulting radicals with hydrogen. This occurs at a temperature of about 350–450 °C and a pressure in the range of 10–20 MPa, as well as in the presence of catalytic systems based on nickel, molybdenum, cobalt, and other metals [45]. It is thanks to the hydrogen environment that it is possible to significantly reduce the formation of coke and secondary heavy residues. Molecules that have just been “broken” by thermal exposure immediately react with hydrogen, turning into lighter, more stable compounds.

Unconventional raw materials, be it oil shale, bitumen sands, or pyrolysis fractions from industrial and household waste, are characterized by an increased content of sulfur, nitrogen, and, often, a significant proportion of high molecular components. Hydrocracking allows for the content of harmful impurities to be reduced to a minimum: under the influence of a catalyst, sulfur compounds are converted into hydrogen sulfide (which is then captured), and nitrogen and oxygen-containing substances are also removed, which improves the quality of the final product. As a result, the resulting liquid fraction (in fact, synthetic oil) is close in properties to classic petroleum feedstock suitable for the production of gasoline, diesel, jet fuel, and other types of fuel [46].

Hydrocracking is used for deep processing of heavy feedstocks such as bitumen, fuel oil, or pyrolysis oil. The process is carried out at temperatures of 300–450 °C and pressures of 5–20 MPa in the presence of hydrogen and catalysts containing platinum group metals (Pt, Pd) or transition metal sulfides (Ni, Mo, Co). During the reaction, carbon bonds are saturated with hydrogen, long chains are split, and sulfur, nitrogen, and oxygen are removed [47].

The most effective catalysts are Ni-Mo/Al_2_O_3_, Co-Mo/Al_2_O_3_, and Pt-ZSM-5, which provide deep desulfurization (99%), high yield of target fractions (up to 95%) and resistance to coke formation [48].

The economic benefits of hydrocracking are that heavy and low-quality resources that are difficult to use can be converted into highly liquid products. On the other hand, the technology requires large capital investments: reactors must withstand high temperatures and pressures, and hydrogen supply systems must be reliable and energy-efficient. In addition, environmental safety issues must be taken into account since hydrogen production is often associated with significant CO_2_ emissions, and catalysts are sensitive to a number of impurities that may be contained in unconventional raw materials [49,50].

Despite these challenges, hydrocracking remains a strategically important technology. As conventional oil resources become depleted and market demand for liquid hydrocarbons remains high, the ability to convert oil shale, bitumen, and even plastic waste into valuable fuels is of particular importance. Add to this the potential for a shift to green hydrogen from renewable energy sources, and hydrocracking could potentially become one of the most environmentally sound technologies, allowing for the efficient use of a variety of unconventional resources while reducing environmental impact.

### 3.4. Catalytic Depolymerization

Catalytic depolymerization is considered today as one of the most promising technologies that allows for obtaining liquid hydrocarbons from a wide variety of non-traditional raw materials—from plastic and rubber waste to some biological residues. Unlike conventional pyrolysis, which requires fairly high temperatures and does not use special catalysts, in the process of catalytic depolymerization, long molecules are broken under the influence of special substances (usually zeolite or metal catalysts) under milder conditions. This not only reduces energy costs but also makes it possible to better control chemical reactions, reducing the yield of by-products and improving the quality of the final fraction.

The essence of the process is that under the influence of a catalyst, polymer raw materials—be it plastic waste, rubber, or even lignocellulosic materials (part of the biomass)—are subject to the rupture of large-molecular bonds, releasing shorter and simpler hydrocarbons. The temperature usually does not exceed 300–350 °C and, sometimes, even occurs at relatively low temperatures (150–200 °C) if effective catalysts and certain conditions of the reaction medium are used. The gas that is formed in parallel with the liquid fraction may contain methane, propane, and butane and serve as an additional source of energy for heating the reactor, and the remaining solid residue is either further processed or utilized.

The process mechanism includes several successive stages. First, chemical bonds in macromolecules are broken, resulting in the formation of shorter hydrocarbon chains and free radicals. Then, thanks to the catalyst, these compounds undergo isomerization and hydrogenation, which improves their stability and increases the proportion of liquid fractions. The next stage involves the removal of oxygen, sulfur, and nitrogen, which makes the final product more suitable for use as fuel. The final stage includes stabilization of the obtained hydrocarbons, reduction of impurity content, and bringing the fractional composition to the desired characteristics [51].

Catalysts play an important role in the efficiency of the process, accelerating reactions and causing them to move in the right direction. Zeolite catalysts are most often used (for example, ZSM-5, Y-zeolites), which contribute to deeper polymer breakdown and an increase in the yield of gasoline and diesel fractions. Metal oxide catalysts (Ni, Co, Mo, ZnO, TiO_2_) help remove unwanted impurities and activate hydrogenation. Bifunctional catalysts (Ni-ZSM-5, Co-Mo/Al_2_O_3_, Fe-Mo/SiO_2_) combine the properties of acid and metal systems, which allows for achieving the maximum yield of liquid hydrocarbons and minimizing coke formation [52].

The main advantage of catalytic depolymerization is its wide flexibility in terms of raw materials. Thus, it is possible to process not only pure plastic streams (for example, polyethylene, polypropylene), but also mixed waste, pre-sorted and crushed. In some cases, even composite materials and multilayer packaging are successfully converted into a liquid fraction similar to oil. After that, the resulting hydrocarbon mixture can be further purified and processed—for example, subjected to hydrotreating or fractionation. Thanks to this, the output product is close in its characteristics to traditional oil, suitable for use in oil refineries. Naturally, this technology has its challenges. Selecting a catalyst is a difficult task since the materials entering depolymerization can have the most unpredictable chemical composition. Plastic, rubber, and various types of biomass contain not only carbon, hydrogen, and oxygen but also a whole range of impurities, including chlorine, sulfur, nitrogen, and metals. These impurities can poison the catalyst and disable it. In addition, catalysts need to be regenerated or replaced periodically, which affects the economic efficiency of the project [53].

However, despite such difficulties, interest in catalytic depolymerization is growing along with the awareness of how large a problem has accumulated in landfills in the form of plastic and other hard-to-decompose waste. If it is possible to reduce the volume of waste and, at the same time, obtain valuable raw materials from it, this has a beneficial effect on both the economy and the environment. Many countries are currently investing in research and pilot projects that improve depolymerization processes and catalysts, hoping that in the future the technology will allow a significant portion of plastics, fabrics, rubber products and other waste to return to the industrial cycle [54].

In the future, when the processes are optimized, catalytic depolymerization will be able to take a worthy place among the methods of processing unconventional hydrocarbon resources, partly replacing the extraction of fossil oil. The resulting liquid fraction, called “synthetic oil”, will prove an important element of a circular economy model in which resources are used more wisely and waste ends up in landfills less often.

### 3.5. Biochemical Methods

Biochemical methods aimed at obtaining synthetic oil from unconventional raw materials are a relatively new direction, actively developing at the intersection of biotechnology, microbiology and the chemical industry. Unlike thermochemical processes based on high-temperature decomposition of organic compounds (pyrolysis, gasification, hydrocracking), biochemical approaches rely on the metabolic capabilities of microorganisms and enzymes capable of converting complex substances into simpler hydrocarbons under relatively mild conditions—moderate temperature and pressure.

These technologies are based on two key mechanisms. The first is associated with anaerobic fermentation when bacteria in an oxygen-free environment decompose organic raw materials (residues of agricultural crops, organic household waste, manure) to form biogas, the main components of which are methane and carbon dioxide. Although biogas is most often burned as a fuel, it can be subjected to further chemical transformations (such as reforming or catalytic conversion) to produce liquid hydrocarbons similar to petroleum [55].

The second mechanism is the use of specially selected or genetically modified microorganisms that are capable of synthesizing hydrocarbon molecules directly from simpler substrates. Such microorganisms (certain strains of algae, bacteria, or yeast) convert sugars, starch, lignocellulose, or other organic compounds into hydrocarbons and their derivatives under the action of enzymes. Sometimes the process takes place in an aqueous medium at moderate temperatures, and the resulting fractions are called microbial oil or bio-oil. Subsequent purification and fractionation make it possible to bring the characteristics of this raw material closer to industrial fuel standards.

The main advantage of biochemical methods is their environmental friendliness and the relative “softness” of the processes. They usually do not require extreme temperatures and pressures, and the aqueous environment in which microorganisms or enzymes work allows us to avoid a number of harmful emissions and reduce energy consumption. In addition, when the feedstock is biomass, we are dealing with a renewable resource, and in the case of using food waste or agricultural waste, we also solve the issue of waste disposal.

However, at present, these technologies are far from mass industrial implementation in the form in which thermochemical processes are implemented. The main reason is the comparatively low productivity and selectivity of biological systems in relation to hydrocarbons: microorganisms and enzymes can be sensitive to fluctuations in the composition of raw materials, temperature conditions, and the presence of toxic impurities. In addition, the collection and preliminary processing of biomass can require costs that reduce profitability. Complex bioreactors are needed to maintain the activity of microorganisms on an industrial scale, and genetic modification to increase the yield of hydrocarbons requires in-depth research and consideration of biosafety [56].

Nevertheless, the prospects for the development of biochemical methods are very promising. Scientists are experimenting with different types of substrates and strains, improving equipment and developing methods for integrating biochemical processes into general processing schemes. For example, excess CO_2_ released at one stage can serve as a nutrient for phototrophic microorganisms (algae), which produce fatty acids and other hydrocarbons during photosynthesis [57]. Also becoming increasingly popular is the “biorefining” approach, when the residues after fermentation are converted into fertilizers or other useful products, improving the overall economic efficiency of the project.

### 3.6. Additional Approaches to Producing Synthetic Oil from Unconventional Hydrocarbon Resources

In addition to the methods already discussed, such as pyrolysis, the Fischer–Tropsch process, and biochemical methods, there are other promising approaches to obtaining synthetic oil and non-hydrocarbon feedstocks. For example, hydrothermal conversion allows for the efficient processing of wet biomass and carbonaceous materials, providing a biocrude yield that can be used as feedstock for synthetic oil. The paper [28] investigates the process of hydrothermal transformation of oil shale with the aim of obtaining synthetic oil and assessing the changes in the pore microstructure occurring in the shale material. The experiments were performed using oil shale samples collected from different deposits. The oil shale was heated to about 350 °C under high pressure (20 MPa). These conditions were chosen to maximize the yield of synthetic oil and to observe changes in the pore structure. The products of the process were evaluated based on their chemical composition. The main focus was on the number of hydrocarbons with a molecular weight of C15–C30, which makes such oils suitable for processing into fuel. Microscopy and porosimetry were used to analyze the pore microstructure, which allowed for a detailed study of changes in porosity and pore distribution in the samples before and after hydrothermal transformation. The results showed that hydrothermal transformation is an effective method for producing synthetic oil with good characteristics. The transformation of oil shale under the selected conditions (350 °C, 20 MPa) provided a yield of synthetic oil up to 30% of the original material weight. This is quite a high figure, especially compared to other shale processing methods. The resulting synthetic oil had a high percentage of hydrocarbons with a molecular weight in the C15–C30 range. This composition is ideal for further processing into fuel, which increases the commercial value of the resulting product. During the conversion, a significant increase in porosity and average surface area of the shale was observed. This is due to the development of micropores and mesopores, which provide improved permeability of the material. These changes in the pore structure help increase the efficiency of shale processing and improve oil yield. The presence of additional pores improves interaction with catalysts and accelerates further processing.

The experiment involved oil shale samples with low permeability coefficients, approximately 0.1 to 0.5 millidarcies [58]. These shales were subjected to in situ combustion, which was carried out in specially prepared boreholes. Air or a mixture of gases was raised into the boreholes to initiate combustion of organic matter, or kerogen, directly in the shale formation. The combustion process was initiated at a temperature of 400 to 600 °C, which is optimal for converting organic material into hydrocarbons. The borehole pressure was maintained at 10–15 MPa, which created stable conditions for successful combustion front propagation. The process was monitored using geophysical methods such as thermography and seismic sounding. These methods allowed for monitoring the combustion front propagation, as well as temperature and porosity changes in the shale formation. The results of the experiment showed that in situ combustion is an effective method for producing synthetic oil. It was possible to obtain up to 25% of synthetic oil from the mass of the original material from low-permeability shales. The chemical composition of the obtained oil included hydrocarbons with a molecular weight in the range of C15–C30, which indicates good suitability of this oil for further processing into fuel. In addition, the combustion process led to the successful propagation of the combustion front along the shale formation over a distance of 5 to 10 m over a period of one to two weeks. This allowed for uniform heating of the formation, which also contributed to better extraction of hydrocarbons. As for the microstructure, the combustion process increased the porosity of the shale by 30–40%. This improvement is due to the thermal destruction of organic matter and minerals, which, in turn, increased the permeability of the rock. As a result, the shales became more suitable for further processing and extraction of hydrocarbons.

The paper [59] focuses on the study of a new method for extracting oil and gas from oil shale. This study used the superheated steam injection pyrolysis method, which allowed us to study the efficiency of this process under real shale formation conditions and evaluate changes in their microstructure. The experimental part of the work used oil shale samples taken from China. The pyrolysis process was carried out in laboratory conditions where oil shale was exposed to superheated steam at different temperatures (from 400 to 600 °C) and pressures (up to 10 MPa). Steam heated to a high temperature was used as a heat carrier, which allowed for the initiation of thermal decomposition of organic matter (kerogen) in the shale material. The pyrolysis process was accompanied by changes in the microstructure of pores and cracks in the shale, which was studied using microscopy and other analytical methods, such as scanning electron microscopy (SEM). These methods allowed us to evaluate the increase in porosity, crack expansion, and changes in the structure of minerals after pyrolysis. The results of the experiment showed that pyrolysis with superheated steam injection is, indeed, effective for extracting oil and gas from oil shale. At 500 °C and 10 MPa, the oil yield was about 15.2% of the original mass, and the gas yield was 5.6%. These data confirm that the superheated steam pyrolysis process can significantly increase the hydrocarbon yield compared to other shale processing methods. In addition, significant changes in the shale microstructure were observed during pyrolysis. The material porosity increased by 30–40%, and the cracks formed during the process expanded, significantly improving the permeability of the shale. This means that after pyrolysis, the shale becomes more suitable for further hydrocarbon extraction since increased permeability facilitates easier extraction of oil and gas.

These methods open up new horizons for the production of synthetic oil from a variety of feedstock sources, providing the possibility of using waste and biomass for energy production.

Comparative characteristics of the considered methods are presented in Table 2.

Thus, modern methods of obtaining synthetic oil from unconventional hydrocarbon raw materials demonstrate a wide range of approaches—from high-temperature thermochemical processes to “soft” catalytic and biochemical technologies. Thermochemical methods (pyrolysis, gasification with subsequent Fischer–Tropsch synthesis) allow for working with a wide range of raw materials—from plastic waste to coal and biomass. However, they require significant energy costs, complex equipment, and high capital investments.

Catalytic methods, such as hydrocracking and catalytic depolymerization, make it possible to obtain high-quality hydrocarbon fractions under more controlled conditions but remain very demanding on the purity and preparation of raw materials, and also require complex infrastructure for supplying hydrogen and maintaining stable operation of catalysts.

Finally, biochemical methods based on fermentation and the use of microorganisms and enzymes have the advantage of low energy consumption and environmental friendliness, allowing for the efficient use of renewable resources and organic waste. However, these processes are still characterized by relatively low speed and difficulties in scaling.

As a result, the choice of the optimal technology is determined by the characteristics of a specific raw material, the availability of infrastructure, economic factors, and environmental priorities. It is likely that many of the listed methods will be used in combination, complementing each other and, thereby, providing the most flexible and efficient way to process unconventional hydrocarbon resources into synthetic oil.

## 4. Catalysts for the Production of Synthetic Oil

### 4.1. Zeolite Catalysts

Zeolite catalysts play a key role in processes aimed at obtaining synthetic oil from unconventional raw materials: plastic and rubber waste, biomass, oil shale, and other carbon-containing materials. Zeolites are aluminosilicate minerals with an ordered porous structure that has a well-defined acid function. Due to the combination of acid centers, the high specific surface area and “shape effect” (shape-selectivity), zeolites provide deep cleavage of macromolecules and the formation of desired hydrocarbon fractions.

Zeolite catalysts have long been considered one of the most effective tools in the production of synthetic oil from various non-traditional types of carbon feedstock: plastics, rubber, biomass, and heavy oil residues. Their high acidity and ordered porous structure allow for cracking and depolymerization processes to be carried out at relatively low temperatures, providing a significant yield of liquid hydrocarbons. At the same time, the formed fractions can be close in quality to traditional oil or high-value petrochemical feedstock.

Research shows that zeolites of different structural types (ZSM-5, Y, β, mordenite, etc.) have different selectivity with respect to target products. Thus, zeolite ZSM-5 (MFI structure) is famous for its ability to form significant amounts of aromatic compounds, including benzene, toluene, and xylenes (BTX). According to the paper of Park et al. [60], during the catalytic pyrolysis of polyethylene (PE) on ZSM-5 at a temperature of about 500 °C, the yield of liquid hydrocarbons increased from 45% (during thermal cracking) to 60–65%, and the content of aromatic components in the liquid fraction reached 25–30%. This result is explained by the acid-catalytic nature of the zeolite where narrow pores stimulate the formation of aromatic structures from branched radicals.

In another study, Kassargy et al. tested the efficiency of HY zeolite in processing mixed plastics (mainly polypropylene) [61]. The scientists found that the use of HY zeolite at 450 °C contributed to the growth of the gasoline fraction (C_5_–C_12_) by 12% compared to the process without a catalyst, and also reduced the formation of waxes and coke. This effect is associated with larger pores of the Faujasite structure of HY, allowing massive macromolecules to penetrate deeper to the active acid sites.

The ultra-stable modification USY (Ultra-Stable Y) deserves special attention. It is used in the cracking of heavy hydrocarbons (e.g., vacuum gas oil) and in the processing of biomass. The study by Liang et al. demonstrated that the introduction of USY zeolite into the fast pyrolysis of wood chips increased the selectivity to light hydrocarbons by 15–20% while reducing the proportion of oxygenated compounds in the crude bio-oil. This improved the energy value of the final product and facilitated subsequent hydrotreating [62].

In addition, beta zeolite (Hβ) and mordenite are also actively studied in the context of waste recycling and synthetic fuel production. Liu et al. used Hβ to pyrolyze mixtures of polyethylene (PE) and polypropylene (PP) waste and recorded a liquid fraction yield of 70%, which significantly exceeds the thermal degradation rates. The scientists attribute such a high yield to the combination of the large-pore structure of the zeolite and increased acidity, which allows for the effective cleavage of polymer chains down to light fractions [63].

Along with obvious advantages (versatility, high selectivity, and the possibility of regeneration), zeolite catalysts have a number of limitations. One of them is the tendency to accumulate coke on the pore surface, which reduces catalytic activity over time. The problem is solved by periodic regeneration in an oxidizing environment at high temperatures (550–600 °C). However, when recycling household plastics, the problem of the presence of chlorine-containing compounds (PVC) or sulfur (in rubber) often arises, which can poison the acidic centers of the zeolite. Some authors point out that preliminary sorting and declaring of raw materials significantly increases the service life of the catalyst [64].

Thus, the use of zeolites, due to their unique microporous structures and high acidity, brings the process of catalytic cracking (pyrolysis) and other methods of processing carbon-containing waste to a higher level of efficiency. The development of pollution-resistant, selective, and easily regenerated zeolite systems remains an important task, opening up prospects for wide industrial application in the field of synthetic oil production.

### 4.2. Metal and Metal Oxide

Metal and metal oxide catalysts play a key role in the processing of non-traditional hydrocarbon raw materials, be it plastics, rubber, biomass, or heavy oil residues. It is due to the presence of active metal centers that it becomes possible to carry out deep splitting (cracking), depolymerization, hydrogenation, and removal of sulfur and nitrogen from the raw materials.

Modern methods of producing synthetic oil from unconventional resources often rely on metal and metal oxide catalysts, each of which has its own “specialization” and unique properties. If we consider the “raw material—catalyst—process conditions” system, the choice of a specific metal or combination of metals is largely determined by the chemical composition and quality of the source material (for example, plastic, rubber, bitumen), as well as the availability of infrastructure (availability of hydrogen, the ability to maintain high pressure or temperature). In the case of nickel catalysts (Ni/Al_2_O_3_) in the pyrolysis of plastics (PE, PP), it is clear that nickel provides enhanced hydrogenation of intermediate radicals [65]. As a result, the proportion of solid wax residues decreases, and the yield of target light hydrocarbons (gasoline and diesel fractions) can increase to 70%. Therefore, where there are high contents of these impurities, preliminary cleaning or sorting of the feedstock is usually required to avoid poisoning the catalyst.

Iron (Fe), used in combination with zeolites (Fe-ZSM-5) [66], is also inexpensive and actively reduces the formation of heavy fractions during pyrolysis. It is important that iron can promote hydrogen transfer reactions, which reduces the risk of coke formation. However, like nickel, it is vulnerable to sulfur. In addition, Fe catalysts often generate more by-products when working with unrefined raw materials, so their “niche” is relatively “clean” waste (for example, mono or binary mixtures of plastic).

If we talk about deep processing of heavy residues (bitumen, vacuum gas oil), then sulfide catalysts based on Co-Mo or Ni-Mo (used on aluminum oxide) prevail on an industrial scale [67]. They are capable of operating at moderate temperatures (350–420 °C) and high pressure (10–15 MPa), providing a high degree of hydrogenation and hydrotreating. As a result, the yield of light distillates (gasoline, diesel) reaches 70–75%, and the sulfur and nitrogen content in the products is significantly reduced. This technology is widely used in oil refineries where the supply of hydrogen and the reaction rate are carefully controlled.

Noble metals—palladium (Pd), platinum (Pt), and ruthenium (Ru)—occupy a separate and more “elite” position due to their exceptional catalytic activity and ability to operate under milder conditions. Pd applied to zeolite HZSM-5 copes well with desulfurization (for example, during the pyrolysis of tires containing sulfur due to vulcanization), and platinum (Pt/Al_2_O_3_) is widely known as a hydrocracking catalyst for obtaining especially pure and stable fuel fractions. However, their high cost and relatively scarce resources make the use of noble metals economically viable only where extreme purification depth or special product properties are required [68,69].

Ruthenium (Ru) has proven itself to be an excellent catalyst in the Fischer–Tropsch reaction, featuring high selectivity for long-chain paraffins and the ability to operate at low temperatures. However, small ruthenium deposits and high price significantly limit its distribution in an industrial format—most often, these are laboratory and pilot studies [70,71].

In general, to summarize, Ni and Fe look more economical and feasible for mass recycling of waste (especially plastics) subject to preliminary sorting, while Co-Mo and Ni-Mo demonstrate stable operation with heavy oil residues and bitumen feedstocks in the presence of hydrogen infrastructure. Pd and Pt are the choice for scenarios where the emphasis is on high-quality end products and the ability to deeply remove sulfur and other harmful components. In turn, Ru remains a niche solution, showing the best results in the synthesis of hydrocarbons from synthesis gas, but it is rarely used on a large industrial scale due to its high cost and rarity [72].

A summary table with examples of the use of metal and metal oxide catalysts in the production of synthetic oil from non-traditional raw materials (plastics, rubber, heavy residues, biomass) is given below (Table 3).

Thus, an analysis of the table with different metal and metal oxide systems shows that no catalyst is universal for any type of feedstock and any conditions. This is why large-scale industrial projects often choose hybrid and multi-stage schemes: for example, pyrolysis of plastic on Ni/Al_2_O_3_ to obtain crude pyrolysis oil, and then, its hydrotreating on a Ni-Mo or Pt catalyst. This approach makes it possible to balance costs, ensure high quality of the final fuel, and solve the problem of waste recycling.

### 4.3. Enzymes for Producing Synthetic Oil

Enzymes are now considered a promising tool for a more “gentle” processing of organic waste and biomass into liquid hydrocarbons, which, with appropriate processing, can become an analogue of synthetic oil. This approach is an alternative to thermochemical methods (pyrolysis, gasification, hydrocracking) and promises a number of environmental and economic advantages, although it still faces a number of technical obstacles.

Unlike high-temperature processes, enzymatic reactions occur at relatively low temperatures (20–60 °C) and atmospheric pressure. Enzymes, which are protein catalysts, are very specific: they accelerate certain reactions, practically without affecting others. Therefore, enzymatic processes for the processing of carbon-containing materials require the selection of specific enzymes or complexes corresponding to the type of raw material that needs to be converted. In the case of plant biomass and agricultural waste, cellulases and ligninases play a key role. The former break down cellulose and hemicellulose into simple sugars, while the latter destroy lignin, a complex polyaromatic polymer that makes wood rigid and hinders its thermal destruction. Similarly, lipases can be used in the processing of fats and oils, which convert triglycerides into fatty acids and glycerol, creating the prerequisites for obtaining hydrocarbons using a subsequent chemical or biochemical stage.

However, the production of “synthetic oil” is not limited to enzymatic decomposition: the products of the initial biocatalytic reactions are most often sugars, organic acids, alcohols, or fatty acids. To bring them to a state close to oil, further chemical processing is often required—for example, hydrotreating and hydrogenation in the presence of metal catalysts (Ni, Co, Pd). This allows us to reduce the proportion of oxygen and sulfur, stabilize the structure of hydrocarbon chains, and increase the yield of the target liquid fraction.

Tomás-Pejó et al. described the processing of wheat straw at 50 °C and pH ~5 with an enzyme cocktail (cellulase + β-glucosidase), where the yield of fermentable sugars reached 70–75% of the theoretical value. Then, yeast (*Saccharomyces cerevisiae*) converted sugars into ethanol and organic acids, and next, the product was partially hydrothermally reduced to an oil-like fraction [74].

One of the priority areas in this field is the creation of genetically modified microorganisms capable of directly synthesizing hydrocarbons (alkanes, alkenes) from simple sugars. Liu and colleagues experimented with genetically modified *E. coli* capable of converting glucose into long-chain alkanes. The yield of final hydrocarbons was still small (less than 10% of the total mass), but the results indicate the possibility of targeted biosynthesis of alkanes [75]. In laboratory studies, strains of *E. coli* have already been obtained that, under certain conditions, form long-chain alkanes that are really close to the components of conventional oil. So far, such yields and concentrations are too low for widespread industrial implementation, but the fact that hydrocarbon biosynthesis is fundamentally possible directly is a serious argument in favor of developing this area.

According to the study by Vescovi et al., enzymatic hydrolysis of edible fats at 35–40 °C using lipase in water resulted in the formation of free fatty acids suitable for subsequent catalytic hydrodexigenation. As a result, some of the products could be used as a biocomponent of diesel fuel. Thus, the role of enzymes is most often focused on the primary “unblocking” of complex macromolecules (cellulose, lignin, fats), facilitating further chemical steps [76].

Despite the obvious advantages, enzymatic methods also face difficulties largely related to their sensitivity to environmental conditions. Enzymes and microorganisms are subject to inactivation in the presence of toxic impurities, heavy metals, some forms of sulfur or chlorine, and also require maintaining a narrow range of pH and temperature. In addition, the rate of enzymatic processes is usually lower than that of thermochemical methods. This means that large reactor volumes and a careful parameter control system are needed to process large volumes of raw materials.

The prospects for improving enzymatic technologies are associated with several factors. The first is the improvement of enzyme immobilization methods on solid carriers (zeolites, silica matrices), which increases their stability and enables multiple use. Secondly, research is expanding on the creation of hierarchical (micro- and mesoporous) matrices where large macromolecules of raw materials can easily penetrate to active centers. And finally, genetic engineering continuously improves microorganisms, “teaching” them to synthesize increasingly complex hydrocarbons from available sugars or organic acids [77].

Thus, the use of enzymes to obtain synthetic oil is an area where biotechnology and classical methods of hydrocarbon processing are organically combined. Although it is still a long way from industrial scale, significant advantages of biocatalysis are already visible today: environmental friendliness (lower energy consumption, less toxic emissions), the potential for processing wet biomass without preliminary drying, high selectivity of reactions. All this makes enzymatic routes for converting organic waste into liquid fuel one of the key areas of “green” chemistry and future energy.

### 4.4. Nanobiocatalysis

Modern research in the field of synthetic oil production demonstrates significant progress due to the introduction of nanobiocatalysts—hybrid systems that combine the principles of nanotechnology and biocatalysis. This approach is based on the use of enzymes or cellular systems fixed on nanostructured carriers, which allows for increasing the selectivity of reactions, accelerating processes, and reducing their energy intensity. Unlike traditional catalysts, nanobiocatalysts allow for processing hydrocarbon raw materials under milder conditions, which is especially important for processing biomass, polymer waste, and hydrocarbon impurities. These technologies can be used as an alternative to traditional processes (pyrolysis, gasification, hydrocracking), and in combination with them to increase the efficiency of raw material processing.

One of the key applications of nanobiocatalysis is the enzymatic decomposition of biomass—wood and agricultural waste containing cellulose, hemicellulose, and lignin. Enzymes such as cellulases and lignases are capable of decomposing these compounds into monomers, which can then be converted into liquid hydrocarbons.

However, the main problem with traditional biocatalysis is the low stability of enzymes and their sensitivity to reaction conditions. To solve these problems, researchers use enzyme immobilization on nanomaterials such as graphene, SiO_2_ nanoparticles, zeolites, and carbon nanotubes [78]. For example, scientists from the Massachusetts Institute of Technology (MIT) have demonstrated that cellulases attached to silicon dioxide (SiO_2_) nanoparticles retain their activity 5 times longer than free enzymes. This opens up the possibility of multiple uses of the biocatalyst, reducing the costs of processing biomass into synthetic fuel [79].

Some microorganisms are capable of directly synthesizing hydrocarbons similar to oil components. To improve their efficiency, scientists use genetic modification and nanotechnology to control metabolic processes. Researchers from the University of California have developed *E. coli* strains capable of converting sugar into hydrocarbons with chain lengths from C5 to C15. This process could become an alternative to petrochemical fuel synthesis, especially in regions with a developed agricultural sector. To increase the yield of hydrocarbons, nanostructured carriers such as metal–organic frameworks (MOFs) are used, which help control metabolic reactions inside microbial cells [80].

The use of nanobiocatalysts in the processing of hydrocarbon feedstock has a number of significant advantages that make this technology a promising alternative to traditional methods such as pyrolysis, gasification, and hydrocracking.

One of the key advantages is the environmental safety of the technology. The use of biocatalysts reduces the need for toxic solvents and aggressive chemical reagents, such as acids or heavy metals, which reduces environmental pollution. In addition, due to milder reaction conditions, fewer by-products are formed, such as polycyclic aromatic hydrocarbons and solid carbon deposits (coke).

Nanobiocatalysis also has high selectivity, which allows for obtaining target products with minimal processing and purification. One of the most significant advantages is the repeated use of nanobiocatalysts, which makes the process more cost-effective [81].

Despite significant advantages, nanobiocatalysis faces a number of challenges that limit its large-scale implementation. One of the main obstacles is the relatively low rate of enzymatic reactions compared to thermochemical methods [7]. For example, the process of gasification or hydrocracking can take from several seconds to minutes, while enzymatic transformations can last hours or even days, which limits their industrial application.

Another difficulty is the high cost of nanomaterials and enzyme immobilization. The production of nanostructured catalysts requires significant costs, especially when using platinum group metals (Pt, Pd, Ru) or complex graphene structures, which increases the cost of the technology [82].

Despite the existing challenges, the development of nanostructured biocatalysts, hybrid enzymatic and chemical processes, as well as new approaches to enzyme immobilization make nanobiocatalysis a promising technology in the field of hydrocarbon processing. In the coming years, we can expect new solutions to integrate nanobiocatalysis with traditional oil refining technologies, making synthetic oil production more sustainable, energy efficient, and environmentally friendly. Immobilization of enzymes on graphene, zeolites, or metal oxide nanoparticles increases their durability, preventing rapid degradation and loss of activity.

## 5. Prospects for the Development of Synthetic Oil

Modern ideas about the future of synthetic oil cover several interrelated areas—from the development of the scientific and technological base to the economic and environmental factors that determine what global energy markets will become in the coming decades.

### 5.1. Practical Application of Synthetic Oil Production Technologies

Modern technological trends in the field of obtaining synthetic oil from unconventional raw materials are multifaceted and are constantly being improved. This process includes both the development of new methods and the improvement of existing approaches, with the main goal remaining to achieve higher efficiency while reducing energy costs and the negative impact on the environment.

In the near future, it is expected that the industry will increasingly combine several methods of processing unconventional raw materials within a single technological complex. For example, if today many companies limit themselves to pyrolysis alone to obtain liquid products from plastic, then projects are already underway where pyrolysis oil is then subjected to hydrocracking or catalytic post-treatment. Such “hybridity” increases the final selectivity to the desired fractions (gasoline, diesel, petrochemical components) and makes it possible to adapt to heterogeneous raw materials—from mixed plastics to household waste and biomass [83].

One of the most significant examples of industrial application of gasification followed by Fischer–Tropsch synthesis is the Shell Pearl GTL project in Qatar. This plant, launched in 2011, is the world’s largest facility for converting natural gas into liquid hydrocarbons, producing up to 140,000 barrels of synthetic fuel per day. The technology is based on the gasification of natural gas to produce synthesis gas (CO + H_2_), which is then catalytically converted into liquid hydrocarbons. This process allows for the production of diesel fuel, aviation kerosene, and chemical components with virtually zero sulfur content, making them more environmentally friendly compared to traditional petroleum products [84].

The main advantage of this project was the use of natural gas as a cheap and stable source of raw materials, as well as the high selectivity of the technology, allowing for the production of premium quality fuel. However, its limitation is high capital intensity—the construction of the plant cost Shell USD 19 billion, which makes such projects available only to large energy companies with strong financial support.

Another example of the successful implementation of modern technologies is the catalytic pyrolysis of plastic waste, which is being actively developed by the American company Brightmark Energy. This company’s plant in Indiana processes up to 100,000 tons of plastic waste per year, converting it into liquid fuel, gaseous hydrocarbons, and synthetic wax [85].

This process allows us to solve several problems at once: recycle plastic waste, reduce the amount of landfills, and obtain energy sources with a relatively low carbon footprint. The use of zeolite catalysts (ZSM-5) makes pyrolysis more selective to liquid hydrocarbons, reducing the yield of gaseous and solid by-products.

However, the technology also has limitations. One of the key factors affecting the efficiency of recycling is the preliminary sorting of the raw material as contaminated plastic or its mixtures with additives reduce the stability of the process. In addition, the high cost of catalysts and the need for their regeneration remain important issues limiting the scaling of this technology.

Not all projects to produce synthetic oil from unconventional feedstocks have been successful. For example, despite significant investments in the development of CTL (coal-to-liquid) technologies in the United States and China, a number of projects have been closed or have significantly limited their activities. In 2015, several American enterprises developing technologies for the direct and indirect conversion of coal into liquid fuel were closed due to falling oil prices, which made such projects economically unprofitable [86].

In China, enterprises using CTL technology have faced strict environmental restrictions associated with high levels of CO_2_ emissions. Coal gasification is accompanied by the release of a significant amount of carbon dioxide, which requires the implementation of expensive CO_2_ capture and storage (CCS) systems, increasing overall production costs [87].

Another example is Recycling Technologies (UK), which was developing catalytic pyrolysis of plastic waste. Despite the technology’s promise, the project ran into financial difficulties in 2022 and was closed [88]. Key challenges included the difficulty of handling raw materials as efficient recycling required the use of pre-cleaned and sorted plastic. In addition, attracting investment proved difficult as the plastic waste recycling market is still in its infancy and faces legislative uncertainty.

A case study of successful and unsuccessful projects shows that technologies for producing synthetic oil from unconventional feedstocks can be economically viable but require significant capital investment and consideration of many factors.

The Shell Pearl GTL and Brightmark Energy projects demonstrate that natural gas gasification and catalytic pyrolysis of plastics can be effective given accessible feedstocks and developed infrastructure. However, CTL projects have faced problems with high CO_2_ emissions and dependence on oil prices, which made them less competitive.

Modern research in the field of nanostructured catalysts and biochemical methods allows us to hope to further improve the efficiency of feedstock processing technologies, reduce energy costs, and improve environmental characteristics. The introduction of carbon capture systems, integration of several processing methods, and improvement of catalysts are key factors that will help make synthetic oil more affordable and environmentally friendly.

### 5.2. Economic Incentives and Scale

The modern development of the synthetic oil industry is largely determined by economic incentives and the possibility of scaling up production. Initially, many synthetic oil production projects seemed like niche experiments, but today, they are increasingly being considered within the framework of national energy security and environmental policy programs.

One of the decisive factors remains world oil prices and the comparability of costs in the production of synthetic fuel. When the price of traditional oil is high, the benefits of alternative methods (coal gasification, waste pyrolysis, shale oil) grow, and the investment interest in them increases. However, with a significant drop in hydrocarbon prices, many synthetic oil projects experience difficulties with payback, which can lead to freezing or postponing their implementation.

Government support measures and environmental regulation are no less important. Many countries have tax breaks, subsidies, or “green” loans that facilitate the launch of projects related to the processing of waste into fuel or the conversion of coal into liquid raw materials. Increasingly strict environmental standards and CO_2_ emission quotas, as well as restrictions on the sulfur content of fuels, are promoting the development of areas related to carbon dioxide capture and storage (CCS) or deep cleaning of feedstocks. Thus, strict environmental legislation indirectly stimulates the growth of the synthetic oil market since the processing of complex and contaminated resources (plastics, tires, bitumen deposits) can be more profitable than paying fines or buying emission quotas [89].

The next aspect is infrastructure and scale. Coal or plastic processing projects require well-established logistics for collecting, sorting, and transporting raw materials, as well as accessible energy infrastructure (hydrogen, powerful power plants, CO_2_ capture systems). In large industrial clusters where several petrochemical and energy facilities can synergistically use each other’s by-products, the cost of synthetic fuels is significantly reduced. At the same time, the scaling possibilities are significantly expanded because large plants always have more potential to reduce specific costs due to the scale effect. Finally, public opinion and ESG factors (environmental, social, governance) are also increasingly influencing investment decisions [90]. The willingness of companies to invest in waste recycling or cleaner oil synthesis technologies often becomes part of their corporate strategy to reduce their carbon footprint and improve their “green” image. On the other hand, if new plants are capable of recycling significant volumes of municipal and industrial waste, this can contribute to the support of the project by authorities and society since the problem of landfills and emissions of plastic fractions into the environment is also solved.

### 5.3. Environmental and Social Aspects

The importance of environmental and social aspects in the design and operation of synthetic oil plants is growing as society increasingly demands less pollution and more sensible use of resources. Technological advances that enable the conversion of plastic waste, biomass, or extra-heavy feedstocks into liquid hydrocarbons must be complemented by measures that ensure minimal negative impact on the environment and take into account the interests of local communities.

The first and, perhaps, the most discussed area is related to waste disposal. Recycling plastics, old tires, and organic residues into synthetic oil significantly alleviates the problem of landfilling. Instead of burning waste or dumping it in landfills, pyrolysis, catalytic depolymerization, and biochemical recycling technologies make it possible to return most of the material to the carbon cycle while obtaining an energy-rich resource. This reduces the load on landfills, reduces the amount of microplastics released into nature, and reduces emissions typical of uncontrolled waste incineration.

One of the most important aspects in organizing such production is the control of emissions and secondary pollution. Although the pyrolysis of plastic or biomass occurs at high temperatures and generally allows to avoid direct combustion, the formation of toxic substances (for example, dioxins in chlorine-containing plastics) or the content of sulfur, chlorine, heavy metals in the feedstock is still possible. To reduce such risks, each enterprise is obliged to implement systems for cleaning gaseous emissions, filtering and capturing carcinogens. Modern catalytic systems and advanced afterburning technologies allow us to minimize harm to air and water to a large extent, but their use significantly increases the cost of the project, which inevitably affects the final economy [91].

The second important block of environmental issues concerns the reduction of the carbon footprint. Even if we talk about the processing of waste or biomass, the final synthetic oil remains a hydrocarbon fuel, the combustion of which produces CO_2_. However, in a situation where the original carbon is “returned” from industrial or biological residues, the overall emissions balance can be much more favorable than when using classic oil from a well, especially if the plant is equipped with carbon dioxide capture systems (CCS) or if we are talking about biomass that has recently absorbed CO_2_ during photosynthesis. Thus, for countries and companies striving for decarbonization, the production of synthetic oil can become an intermediate link in a more environmentally friendly energy balance, especially where it is impossible to quickly switch to electricity or hydrogen (for example, in aviation). From a social point of view, issues of safety and the impact on the local population and jobs have a great resonance. The creation and operation of waste-to-fuel synthesis plants can become a driver for regional development, providing employment and forming a new special infrastructure (sorting stations, logistics centers). At the same time, local residents often express concerns about possible air or water pollution and the appearance of unpleasant odors. Therefore, companies are forced to build a dialogue with the public: implement transparent emission monitoring, ensure environmental audits, publish data on the composition of emissions, and comply with strict rules for the storage and transportation of raw materials [92,93].

Finally, we cannot ignore the issue of resource conservation. Using not only traditional oil fields but also secondary sources of carbon—plastics, tire dust, agricultural residues—we distribute available resources more wisely, striving for a circular economy. This approach reduces the risk of rapid depletion of classic oil deposits and, also, reduces dependence on geopolitical factors affecting prices and access to hydrocarbons.

Thus, the environmental and social aspects of synthetic oil production acquire a dual focus. On the one hand, this is a solution to problems: waste disposal, emissions regulation, reduction of CO_2_ volumes generated by classic waste incineration or oil production. On the other hand, these are new challenges associated with the need to implement expensive cleaning systems, comply with strict environmental standards, and establish trust with the local population. If these factors are properly taken into account, synthetic oil can become an important link in the “green” transformation of energy, integrating into the concept of a closed cycle where waste becomes not ballast but a full-fledged resource.

## 6. Conclusions

A study of the possibilities and practical approaches to obtaining synthetic oil from unconventional hydrocarbon raw materials demonstrates that this area is developing under the influence of a whole range of technological, economic, environmental, and social factors. On the one hand, excessive accumulation of waste, depletion of “easy” oil fields, and the increased need to diversify hydrocarbon sources create an objective need for innovative methods of processing plastics, tires, biomass, coal, and other complex or difficult-to-utilize raw materials. On the other hand, modern technological advances, including multi-stage processes (pyrolysis, gasification, hydrocracking, catalytic depolymerization), catalyst improvement, and integration of enzymatic and chemical stages, as well as the introduction of CO_2_ capture and storage systems (CCS), allow for achieving high efficiency and increasingly deeper processing of raw materials.

Economic incentives arise due to fluctuations in prices for traditional oil and environmental legislation that encourages waste recycling and carbon footprint reduction. At the same time, increasing the volume of synthetic oil production requires scaling up the infrastructure for collecting, sorting, and transporting raw materials, as well as developing industrial clusters where internal cooperation between different industries is established.

Environmental and social aspects occupy a special place in this industry. Waste recycling (plastics, tires, organic waste) provides a direct solution to the problem of its accumulation in landfills and release into the environment but requires control over toxic emissions and the organization of a safe cycle for the disposal of products and by-products. The use of “green” technologies and bio-raw materials, as well as CCS systems, allows for a partial or significant reduction in the carbon footprint of the final fuel. The social significance of projects increases due to the creation of new jobs, solving the problem of garbage and involving the population in environmentally oriented programs. At the same time, a dialogue with local residents, ensuring transparent monitoring of emissions and adherence to high environmental standards, is a prerequisite for strengthening trust and the successful development of the industry.

Thus, obtaining synthetic oil from unconventional hydrocarbon raw materials appears to be a promising direction, combining technological achievements with real ecological and economic benefits. Strengthening state support, improving scientific approaches (from the development of nanocatalysts to the use of biotechnology), increasing the number of large integrated projects, and taking into account the social agenda can collectively transform this industry from a high-tech niche into an important component of modern energy and industry.

## Figures and Tables

**Table 1:** Feedstock for producing synthetic oil.

Type of Raw Material	Advantages	Disadvantages	Potential for Oil Synthesis
Oil shale and bitumen sands	- Large proven reserves - Possibility of obtaining heavy fractions close to oil - Proven (albeit energy-intensive) technologies for extraction and processing	- High extraction and processing costs - Large volumes of waste and high-water consumption - Serious environmental impact (landscape disturbance, greenhouse gas emissions)	- A significant source of alternative oil at high prices for raw materials - Improvements in environmental and energy-efficient methods of extraction are required - Can become a strategic reserve for countries with large deposits
Biomass	- Renewable and widely available - Possibility of recycling agricultural and organic waste - Lower carbon footprint	- Low yield of liquid hydrocarbons without additional stages (catalytic processing) - Possible competition with food farming - Seasonality and heterogeneity of raw materials	- Promising source of “green” fuels - Pyrolysis, gasification (Fischer–Tropsch synthesis), hydrothermal processing technologies are being actively improved - High potential when integrated with waste processing complexes
Coal	- Large global reserves - Long-known conversion technologies (gasification, direct hydrogenation) - Possibility of obtaining high-quality fuel	- High energy and water costs during liquefaction - Significant CO_2_ emissions during combustion and processing - Environmental problems with mining (especially open-pit mining)	- With favorable oil prices, it can be economically viable - Development of CO_2_ capture and storage (CCS) technologies can make the process more environmentally friendly - Promotes energy independence for countries with large coal deposits (e.g., China, USA, Russia)
Industrial and domestic waste	- Double benefit: solving the disposal problem and obtaining liquid fuel - Reducing the volume of waste disposal - Reducing greenhouse gas emissions	- Need for sorting and preliminary preparation of raw materials - Dependence of efficiency on the composition and regularity of waste receipt - Costly technologies (pyrolysis, gasification, catalytic depolymerization)	- A promising area for the implementation of closed-loop technologies (circular economy) - Can be effectively developed with the tightening of environmental legislation - Of particular interest is the processing of plastics and rubber (high carbon content)

**Table 2:** Comparative characteristics of the methods.

Method	Raw Materials and Preparation	Key Stages	Advantages	Disadvantages
1. Pyrolysis	- Plastics, rubber, biomass, oil shale - Often requires crushing and drying	Heating to 400–600 °C (or higher) Absence of oxygen Decomposition of macromolecules into gas, pyrolysis oil, and coke	- Universality in raw materials (suitable even for complex and contaminated waste) - Possibility to reduce the volume of landfill waste - Relatively simple reactor type	- High energy consumption (requires significant heating) - The resulting “pyrolysis oil” requires deep purification and processing - Possible formation of toxic substances that require capture
2. Gasification + synthesis (Fischer-Tropsch method)	- Coal, biomass, waste, heavy–residues - Pre-crushing, drying (if necessary)	Gasification at 800–1500 °C (partial oxidation/steam) → synthesis gas (CO + H_2_) Purification of synthesis gas (removal of S, CO_2_) Fischer–Tropsch reaction at 200–350 °C and high pressure	- Flexibility in raw materials (coal, biomass, waste) - High quality products (low sulphur content, etc.) - Possibility of large-scale production	- Complex and expensive technology (gasifier + synthesis) - Requires thorough purification of synthesis gas, complex catalysts - Profitability depends on scale and energy prices
3. Hydrocracking	- Heavy oil residues, shale oil, bitumen, pyrolysis oil - Pre-stabilization is often required	Heating to 350–450 °C at a pressure of 10–20 MPa Hydrogen supply + catalyst (Ni-Mo, Co-Mo) Splitting molecules with simultaneous saturation with hydrogen	- High yield of light fractions (gasoline, diesel) - Improved quality (reduction of sulfur, nitrogen) - Minimal coke formation	- Requires high pressure and hydrogen (complex, expensive equipment) - Sensitivity of the catalyst to sulfur and other impurities - Dependence on hydrogen sources (usually “gray”)
4. Catalytic depolymerization	- Plastic waste (PE, PP), rubber, part of biomass - Pre-sorting, cleaning from non-carbon inclusions	Heating to 150–350 °C, catalyst (zeolites, metals) Breaking polymer chains under milder conditions Separation of liquid and gas fractions	- Mild conditions compared to thermal pyrolysis (below T) - Output of liquid close to petroleum hydrocarbons - Processing of plastics and other polymers	- Raw materials require sorting and cleaning (catalysts are “afraid” of contamination) - The technology is in an active stage of development; large volumes are not always achievable - Catalysts can quickly deactivate
5. Biochemical methods	- Biomass (plant, food waste), microalgae, organic residues - Often requires pre-fermentation or medium preparation	Anaerobic digestion (production of biogas) or targeted biosynthesis by microorganisms Conversion of biogas or “bio-oil” fractions into liquid fuel Cleaning and conditioning	- Low energy consumption (processes at moderate T, atmospheric pressure) - Environmentally friendly, use of renewable resources - Potentially closed cycle (CO_2_ → microorganisms → hydrocarbons)	- Low productivity (processes are slower than thermochemical ones) - Difficulty of scaling: microorganisms are sensitive to contamination and changing conditions - High costs of maintaining bioreactors, complexity of genetic modification
6. Hydrothermal conversion	Bitumen, shale, water	Thermal decomposition with water	Low temperature efficiency, environmentally friendly	Products require processing, difficult to control
7. In situ combustion	Low permeability shale	In situ combustion, crack expansion	High efficiency, handles difficult deposits	High capital costs, contamination risks
8. Superheated steam injection pyrolysis	High kerogen shale	Pyrolysis with superheated steam injection	High hydrocarbon yield, energy saving	High equipment costs, temperature and pressure requirements

**Table 3:** Examples of the use of metal and metal oxide catalysts in the production of synthetic oil.

Catalyst	Raw Materials/Process	Basic Conditions	Key Results/Effects	Sources
Ni/Al_2_O_3_	Pyrolysis of polyethylene (PE)	T = 350–450 °C	- Reduced wax yiel - Increased liquid fraction up to ~70% - Less coke formation	[65]
Fe-ZSM-5	Pyrolysis of mixed plastics	T = 450–500 °C	- +10–15% to the gasoline fraction (C_6_–C_12_) - Reduction of heavy residue - Accelerated polymer breakdown	[66]
Co-Mo/Al_2_O_3_ (sulfides)	Hydrocracking of bitumen residues	T = 350–420 °C, P = 10–15 MPa	- Deep desulfurization - Yield of light distillates ~70–75% - Reduction of nitrogen and resins	[67]
Pd/HZSM-5	Pyrolysis of tire rubber	T = 400–450 °C	- Reduction of sulfur compounds - +10–15% to the share of aromatic hydrocarbons - Increased process stability	[68]
Pt/Al_2_O_3_	Hydrocracking of heavy oil residues	T = 350–400 °C, P = 10–20 MPa	- High conversion depth - Lower reaction temperature - Improved quality of final fuel (less sulfur)	[69]
Ru (on different supports)	Fischer–Tropsch method from synthesis gas	T = 200–220 °C, P = 10–20 MPa	- Maximum selectivity to long-chain paraffins - High activity at low T - Rare industrial application (due to price)	[70,71]
Ni-Mo/Al_2_O_3_ (oxides/sulfides)	Hydrocracking of bitumen, heavy residues	T = 350–400 °C, P = 10–15 MPa	- Output of diesel/gasoline fractions up to 70–75% - Removal of sulfur, nitrogen - Requires a high-quality hydrogen environment	[72]
Ni-ZSM-5 (bifunctional)	Co-pyrolysis of PE and PP	T = 350–400 °C	- Up to 75% liquid fraction - High aromatic content - Use of gas fraction for heating the reactor	[73]

## Data Availability

Not applicable.
